# A Universal Rank-Size Law

**DOI:** 10.1371/journal.pone.0166011

**Published:** 2016-11-03

**Authors:** Marcel Ausloos, Roy Cerqueti

**Affiliations:** 1 School of Business, University of Leicester, University Road, Leicester, LE1 7RH, United Kingdom; 2 GRAPES – Group of Researchers for Applications of Physics in Economy and Sociology, Rue de la Belle Jardiniere 483, B-4031, Angleur, Belgium; 3 University of Macerata, Department of Economics and Law, Via Crescimbeni 20, I-62100, Macerata, Italy; East China University of Science and Technology, CHINA

## Abstract

A mere hyperbolic law, like the Zipf’s law power function, is often inadequate to describe rank-size relationships. An alternative theoretical distribution is proposed based on theoretical physics arguments starting from the Yule-Simon distribution. A modeling is proposed leading to a universal form. A theoretical suggestion for the “best (or optimal) distribution”, is provided through an entropy argument. The ranking of areas through the number of cities in various countries and some sport competition ranking serves for the present illustrations.

## 1 Introduction

Approaches of hierarchical type lie behind the extensive use of models in theoretical physics [1], the more so when extending them into new “applications” of statistical physics ideas [2, 3], e.g. in complex systems [4] and phenomena, like in fluid mechanics [5, 6] mimicking agent diffusion. In several studies, researchers have detected the validity of power laws, for a number of characteristic quantities of complex systems [7–11]. Such studies, at the frontier of a wide set of scientific contexts, are sometimes tied to several issues of technical nature or rely only on the exploration of distribution functions. To go deeper is a fact of paramount relevance, along with the exploration of more grounding concepts.

The literature dealing with the rank-size rule is rather wide: basically, papers in this field discuss why such a rule should work (or does not work). Under this perspective, Pareto distribution and power law, whose statement is that there exists a link of hyperbolic type between rank and size, seem to be suitable for this purpose. In particular, the so-called first Zipf’s law [8], which is the one associated to a unitary exponent of the power law, has a relevant informative content, since the exponent can be viewed as a proxy of the balance between outflow and inflow of agents.

**Fig 1:**
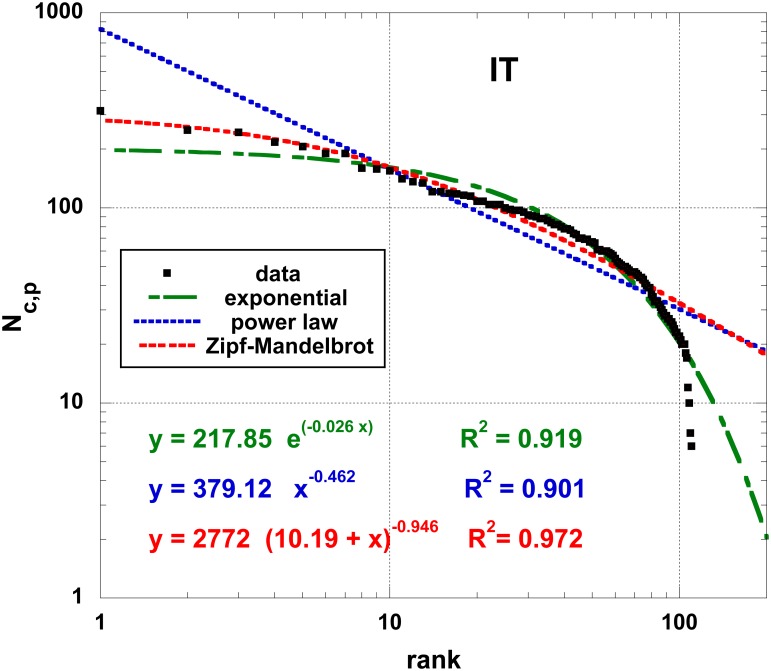
Relationship between the number *N*_*c*,*p*_ of IT cities (8092) per provinces (110) on a log-log scale. The ranking criterion is the one associated to the number of cities (high rank when the number of cities is high). The reference year is 2011. Several fits are shown: power law, exponential and Zipf-Mandelbrot function, Eq (1). The corresponding correlation coefficients are given; different colors and symbols allow to distinguish cases.

The theoretical explanation of the Zipf’s law has been the focus of a large number of important contributions [12–17]. However, the reason on why Zipf’s law is found to be a valid tool for describing rank-sizes rule is still a puzzle. In this respect, it seems that no theoretical ground is associated to such a statistical property of some sets of data [18, 19]. Generally, Zipf’s law cannot be viewed as a universal law, and several circumstances rely on data whose rank and size relationship is not of hyperbolic nature. Such a statement is true even in the urban geography case,—the one of the original application of the Zipf’s law, for the peculiar case of cities ranking. Remarkable breakdowns has been assessed e.g. in [20–25]. A further example is given by the number *N*_*c*,*p*_ of cities (*c*) per provinces (*p*) in Italy (∑_*c*,*p*_ *N*_*c*,*p*_ = 8092), see the log-log plot of the data from 2011 in Fig 1: the (110) provinces are ranked by decreasing order of “importance”, i.e. their number of cities. Fits by (i) a power law, (ii) an exponential and (iii) a Zipf-Mandelbrot (ZM) function [26]

$$y\left(r\right)=\hat{c}/{\left(\rho +r\right)}^{\omega }\;\equiv \;{\left[c/\left(\rho +r\right)\right]}^{\omega },$$ (1)

*r* being the rank.

The fits are, the least to say, quite unsatisfactory in particular in the high rank tail, essentially because data usually often presents an inflection point.

Therefore, no need to elaborate further that more data analysis can bring some information on the matter.

The paper is organized as follows. In Section 2, an alternative to a hyperbolic rank-size law and its above “improvements” are discussed: the data can be better represented by an (other than Zipf’s law) analytic empirical law, allowing for an inflection point.

Next, we introduce a universal form, allowing for a wider appeal, in Sect. 2.2, based on a model thereafter presented in Sect. 2.3. Such a general law can be turned into a frequency or probability distribution. Thus, the method suggests to consider a criterion of possibly optimal organization through the notion of relative distance to full disorder, i.e., a ranking criterion of entity distributions based on the entropy (Section 2.3). Section 3 allows us to conclude and to offer suggestions for further research lines.

## 2 An alternative to a hyperbolic rank-size law

In the context of best-fit procedures, rank-size theory allows to explore the presence of regularities among data and their specified criterion-based ranking [27]. Such regularities are captured by a best-fit curve. However, as observed in Fig 1, the main problem strangely resides in missing the distribution high rank tail behavior. No doubt, that this partially arises because most fit algorithms take better care of the high values (on the *y*-axis) than the small ones. More drastically, a cause stems in the large rank *r* tail which is usually supposed to extend to infinity, see Eq (2), but each system is markedly always of finite size. Therefore, more complicated laws containing a power factor, like the stretched exponential or exponential cut-off laws should be considered inadequate.

We emphasize that we are in presence of data which often exhibits an inflection point.

The presence of an inflection point means that there is a change in the concavity of the curve, even if the slope remains with the same (negative) sign for the whole range. Thus, one could identify two regimes in the ranked data, meaning that the values are clustered in two families at a low and high ranks. In such cases, the finite cardinality *N* of the dataset leads to a collapse of the upper regime at rank *r*_*M*_ ≡ *N*. Nevertheless, the Yule-Simon distribution [28],

$$y\left(r\right)=d\;r^{-\alpha }\;e^{-\lambda r},$$ (2)

could be arranged in an appropriate way, according to a Taylor expansion as in [24]. Eq (2) can be then rewritten as

$$y\left(r\right)=\kappa_{3}\;\frac{{\left(N\;r\right)}^{-\gamma }}{{\left(N-r+1\right)}^{-\xi }},$$ (3)

as discussed by Martinez-Mekler et al. [29] for rank-ordering distributions,—in the arts and sciences; see also more recent work on the subject [30] with references therein. This Eq (3) is a (three-parameter) generalization of

$$y\left(r\right)=K\;{\left(\frac{N\cdot r}{N-r+1}\right)}^{-\beta }\;\equiv \;\kappa_{2}\;{\left(\frac{r}{N-r+1}\right)}^{-\beta },$$ (4)

the (t-parameter) function used when considering the distribution of impact factors in bibliometrics studies [31–35], i.e., when *γ* ≡ *ξ*, and recently applied to religious movement adhesion [36]. Notice that there is no fundamental reason why the decaying behavior at low rank should have the same exponent as the collapsing regime at high rank: one should *a priori* admit *γ* ≠ *ξ*.

**Fig 2:**
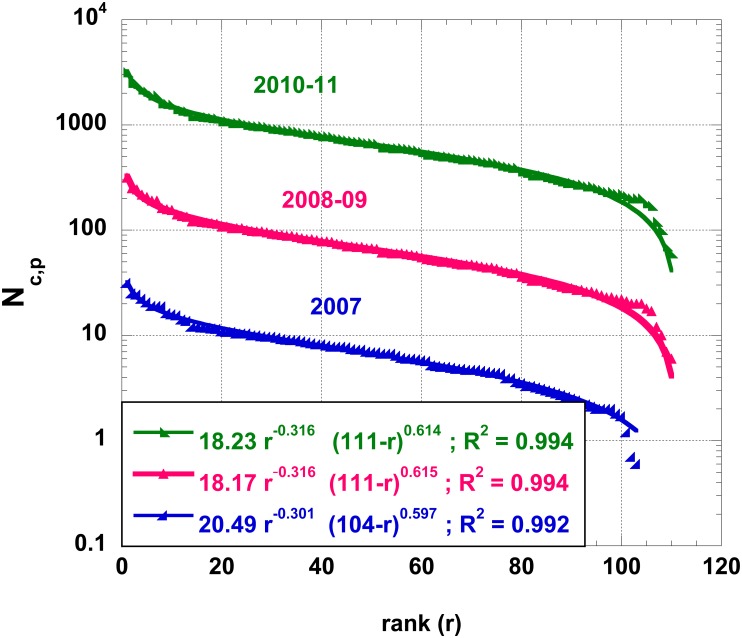
Semi-log plot of the number of cities in IT provinces, *N*_*c*,*p*_; the provinces are ranked by their decreasing “order of importance”, for various years; the 2007, 2008–2009 and 2010–2011; data are displaced by an obvious factor for better readability; the best 3-parameter function, Eq (3), fit is shown. Parameter values are obtained by fits through Levenberg-Marquardt algorithms with a 0.01% precision.

In fact, such an alternative law is easily demonstrated to be an appropriate one for describing size-rank data plots. For example, reconsider the IT *N*_*c*,*p*_ case, shown in Fig 1, redrawn on a semi-log plot in Fig 2. (All fits, in this communication, are based on the Levenberg-Marquardt algorithm [37–39] with a 0.01% imposed precision and after testing various initial conditions for the regression process.) The rank-size relationship appears to follow a flipped noid function around some horizontal mirror or axis. Notice that similar behaviors are observed for different years, although the number of *N*_*c*,*p*_ yearly differs. Incidently, note that, in this recent time, the official data claims a number of 103 provinces in 2007, with an increase by 7 units (BT, CI, FM, MB, OG, OT, VS, in conventional notations) thereafter, leading to 110 provinces. The number of municipalities has also been changing, between 2009 and 2010, whence the rank of a given province is not constant over the studied years.

### 2.1 Other illustrating topics and generalization

In view of taking into account a better fit at low and high rank, one can generalize Eq (3) to a five parameter free equation

$$y\left(r\right)=\kappa_{5}\;\frac{{\left(N\;\left(r+\Phi \right)\right)}^{-\gamma }}{{\left(N+1-r+\Psi \right)}^{-\xi }},$$ (5)

where the parameter Φ takes into account Mandelbrot generalization of Zipf’s law at low rank, see Eq (1), while Ψ allows some flexibility at the highest rank.

In particular, the shape of the curve in Eq (5) is very sensitive to the variations of Φ and Ψ.

As the parameter Φ increases, the relative level of the sizes at high ranks is also increased. This means that the presence of outliers at high ranks is associated to high values of Φ. If one removes such outliers from the dataset and implements a new fit procedure, one obtains a lower level of the calibrated Φ and a flattening of the curve at low ranks. In [40], the authors have found something similar in a different context; they have denoted the major (upsurging) outlier at rank 1 by “king” and called the other outliers at ranks 2, 3, … as “viceroys”. The removal of outliers necessarily leads to a more appealing fit, in terms of visualization and *R*^2^, when such a procedure is implemented through power laws. In this respect, the introduction of a further parameter—Φ, in this case—serves as adjustment term at high ranks, and represents an improvement of the previous theory.

Indeed, the parameter Ψ acts analogously to Φ, but at a low rank. In particular, an increase of Ψ is associated to a flattening of the five parameter curve of Eq (5) at medium and low ranks. Such a flattening is due to sizes at low ranks which are rather close to those at medium ranks. This phenomenon has been denoted in [41] as “queen” and “harem” effect,—to have in mind the corresponding “king” and “viceroys” effects at low ranks. The queen and harem effect is responsible of the deviations of the power law from the empirical data at a low rank. Thus, the parameter Ψ also constitutes an adjustment term at low ranks and is an effective improvement of the performance of the fitting procedure.

Substantially, the specific sense of Ψ should be also read in terms of “generalization” and “in view of best fit”. Usually, one is not sure about the 0 at the origin of axes. Our Φ corresponds to the *ρ* of Mandelbrot (see Eq (1)), for which Mandelbrot gives no interpretation: it is only a mathematical trick. Thus, by “symmetry”, we introduce a Ψ at high rank. It allows some flexibility due to possible sharp decays, due to outliers at high ranks. This also allows to move away from strict integers, and open the functions to continuous space as done in Sect. 2.2.

**Fig 3:**
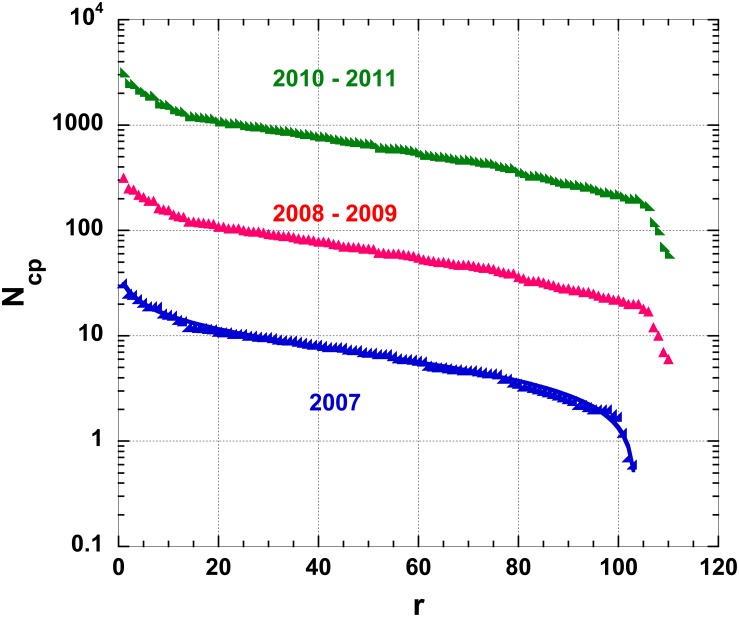
Semi-log plot of the number of cities in IT provinces, *N*_*c*,*p*_; the provinces are ranked according to their decreasing “order of importance”, for various years; the 2007 and 2010–2011 data are displaced by an obvious factor of 10 for better readability; the best 5-parameter function, Eq (5), fit is shown. Parameter values are obtained by fits through Levenberg-Marquardt algorithms with a 0.01% precision.

We have compared the fits conceptualized in Eqs (3) and (5) for the specific IT *N*_*c*,*p*_ case (compare Figs 2 and 3 and Table 1). Even if both these laws are visually appealing and exhibit a high level of goodness of fit, the *R*^2^ associated to Eq (3) is slightly lower than that of Eq (5). Thus, we can conclude that the five-parameters law, Eq (5), performs better than the three-parameters one, Eq (3).

**Table 1:** Best fit parameters and *R*^2^ for the number of IT cities in various years, for either cases, i.e. Eqs (3) and (5). The parameters pertain to the displaced data as visualized on Figs 2 and 3.

Eq (3)			
	2007	2008/2009	2010/2011
*N*	103	110	110
*κ*_3_ *N*^−*γ*^	2.049	18.177	182.265
*γ*	0.301	0.316	0.316
*ξ*	0.597	0.615	0.614
*R*^2^	0.99240	0.99445	0.99441
Eq (5)			
	2007	2008/2009	2010/2011
*N*	110	110	110
*κ*_5_ *N*^−*γ*^	3.971	33.709	332.71
*γ*	0.373	0.387	0.386
*ξ*	0.499	0.527	0.529
Ψ	-7.441	0.608	0.640
Φ	0.945	0.926	0.906
*R*^2^	0.99402	0.99631	0.99623

Even though one could display many figures describing the usefulness of the above, let us consider two cases, e.g. in sport matter.

**Fig 4:**
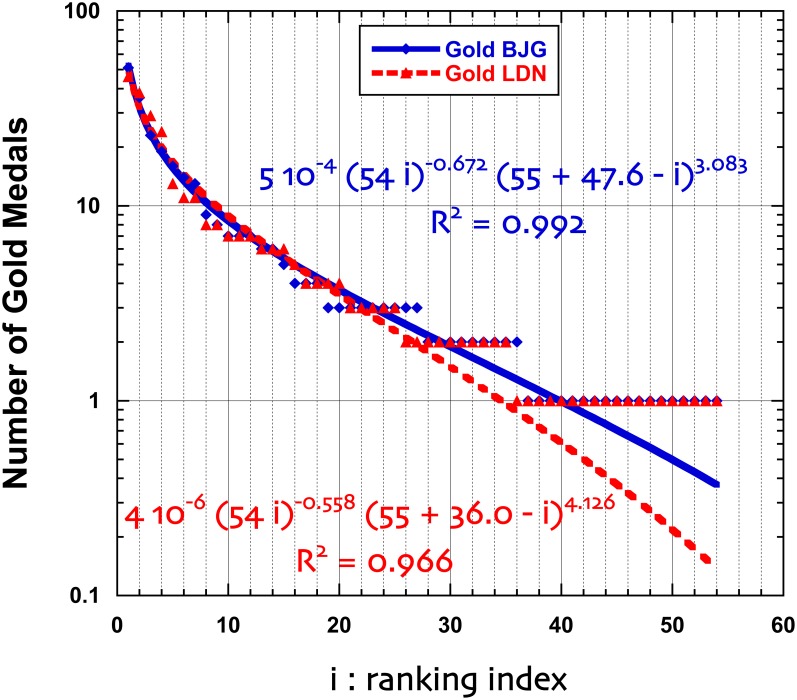
Semi-log plot of the number of Gold medals obtained by countries at Beijing (BJG) and London (LDN) recent Summer Olympic Games, as ranked according to their decreasing “order of importance” index *i*; the best 4-parameter fitting function is displayed, Eq (5), with Φ = 0.

**Fig 5:**
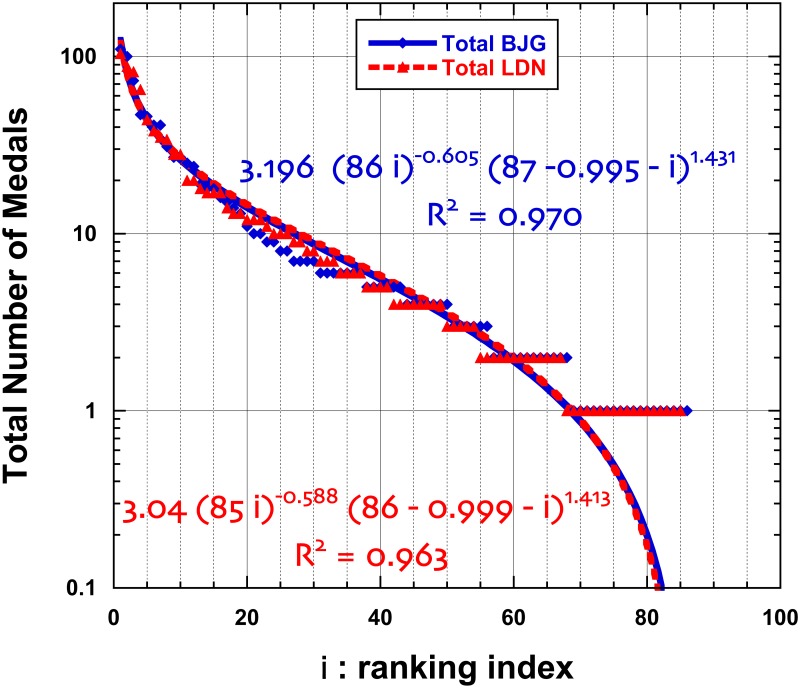
Semi-log plot of the total number of medals obtained by countries at Beijing (BJG) and London (LDN) recent Summer Olympic Games, as ranked according to their decreasing “order of importance” index *i*; the best 4-parameter fitting function is displayed, Eq (5), with Φ = 0.

**Fig 6:**
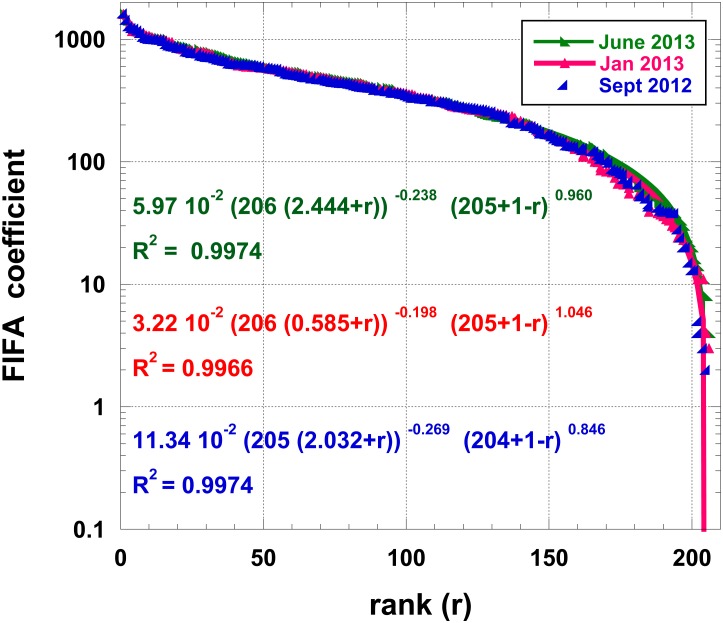
Semi-log plot of the FIFA countries ranked by their decreasing “order of importance” through the FIFA coefficient; the best 5-parameter function, Eq (5), is shown.

- Consider the ranking of countries at recent Summer Olympic Games: Beijing 2008 and London 2012. The ranking of countries is performed trough the number of “gold medals”, but one can also consider the total number of medals,—thus considering a larger set of countries. A country rank is of course varying according to the chosen criterion. It is also true that due to subsequent analysis of athlete urine and other doping search tests, the attribution of medals may change with time. We downloaded the data available on Aug. 13, 2012, from http://www.bbc.co.uk/sport/olympics/2012/medals/countries Interestingly, the number of gold medals has not changed between Beijing and London, i.e. 302, but due to the “equivalence of athletic scores”, the total number of medals is slightly different: 958 → 962. Moreover, the number of countries having received at least a gold medal is the same (54), but the total number of honored countries decreased from 86 to 85. Obviously, in contrast to the administrative data on IT provinces ranking, there is much “equality between countries” in Olympic Games; therefore a strict rank set contains many empty subsets. It is common to redefine a continuous (discrete) index *i* in order to rank the countries. Moreover, the rank distributions are much positively skewed (skewness ∼ 3) with high kurtosis (≥10). Therefore, the inflection points occur near *r* = *r*_*M*_/2 and for a size close to the median value. On Figs 4 and 5, such a ranking for Olympic Games medals is displayed, both for the Gold medal ranking and the overall (“total”) medal ranking. Reasonably imposing Φ = 0, the parameters of Eq (5) lead to remarkable fits, even though the collapsing behavior of the function occurs outside the finite *N* range. We have tested that a finite Φ does not lead to much regression coefficient *R*^2^ improvement.
- In other sport competitions, the “quality” of teams or/and countries is measured through quantities which are not discrete values. For example, in soccer, more than 200 federations (called “Association Members”, ∼ countries) are affiliated to the FIFA (http://www.fifa.com/worldranking/procedureandschedule/menprocedure/index.html). The FIFA Country ranking system is based on results over the previous four years since July 2006. It is described and discussed in [42] to which we refer the reader for more information. Note that a few countries have zero FIFA coefficients. Interestingly the skewness and kurtosis of the FIFA coefficient distributions are rather “well behaved” (close to or ≤1.0), while the coefficient of dispersion is about 250. From previous studies, it can be observed that the low rank (“best countries”) are well described by a mere power law, including the Mandelbrot correction to the Zipf’s law. However, the high tail behavior is poorly described. We show in Fig 6 that the generalized equation is much better indeed. From a sport analysis point of view, one might wonder about some deviation in the ranking between 170 and 190.

### 2.2 Universal form

These displays suggest to propose some universal vision as presented next.

It is easily observed in Eq (3) that a change of variables *u* ≡ *r*/(*N* + 1), leads to

$$y_{1}\left(u\right)=\hat{\kappa_{3}}\;\left[u^{-\gamma }{\left(1-u\right)}^{\xi }\right]$$ (6)

However, in so doing, *u* ∈ [1/(*N* + 1), *N*/(*N* + 1)].

In order to span the full [0, 1] interval, it is better to introduce the reduced variable *w*, defined as *w* ≡ (*r* − 1)/(*r*_*M*_ − 1), where *r*_*M*_ is the maximum number of entities. Moreover, in order to fully generalize the empirical law, in the spirit of ZM, Eq (1), at low rank, a parameter *ϕ* can be introduced. In the same spirit, we admit a fit parameter *ψ* allowing for possibly better convergence at *u* ≃ 1; we expect, *μ* ∼ 1/*r*_*M*_.

Thus, we propose the universal form

$$y_{2}\left(w\right)=\eta \;\;{\left(\phi +w\right)}^{-\zeta }\;\;{\left[1-w+\psi \right]}^{\chi },$$ (7)

for which the two exponents *χ* and *ζ* are the theoretically meaningful parameters. The amplitude *η* represents a normalizing factor, and can be then estimated. Indeed, by referring to the case *χ* ∈ (0,+∞) and *ζ* ∈ (0, 1) and posing $\tilde{w}=\phi +w$ and *u* = 1 + *ϕ* + *ψ*, we can write

$$\begin{array}{c} \eta ={\left[\int_{{\tilde{w}}_{0}}^{{\tilde{w}}_{1}}{\tilde{w}}^{-\zeta }{\left(u-\tilde{w}\right)}^{\chi }\;d\tilde{w}\right]}^{-1}\equiv \\ \frac{1}{{\left(1+\phi +\psi \right)}^{1+\chi -\zeta }}\;\;\frac{1}{{\left[B_{t}\left(1-\zeta ,1+\chi \right)\right]}_{t_{0}}^{t_{1}}}\;\;, \end{array}$$ (8)

with *t*_0_ = *ϕ*/(1 + *ψ* + *ϕ*), and *t*_1_ = (1 + *ϕ*)/(1 + *ψ* + *ϕ*), and where *B*_*t*_(*x*, *y*) is the incomplete Euler Beta function [43–45], itself easily written, when *t* = 1, in terms of the Euler Beta function,

$$B\left(x,y\right)\equiv B_{1}\left(x,y\right)=\frac{\Gamma (x)\Gamma (y)}{\Gamma (x+y)};$$ (9)

Γ(*x*) being the standard Gamma function.

**Fig 7:**
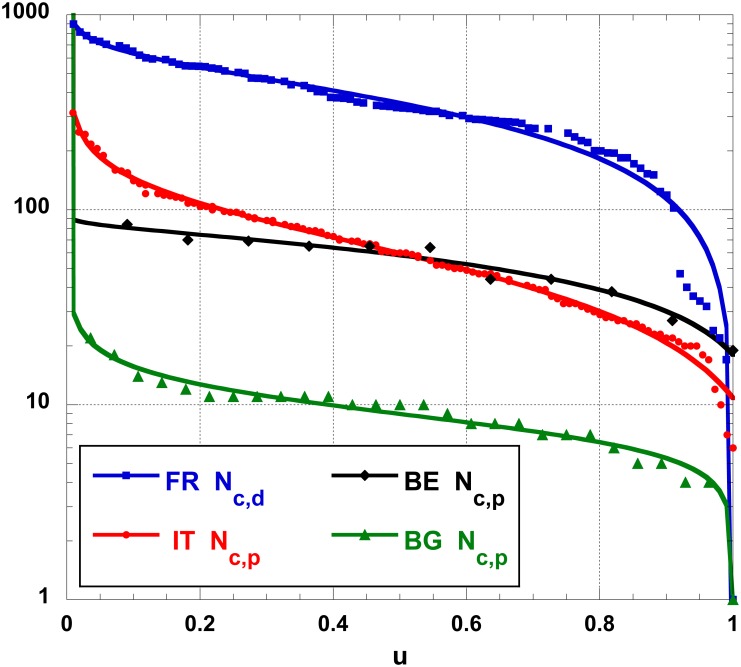
Semi-log plot of the number of cities, *N*_*c*,*p*_ and *N*_*c*,*d*_, ranked by decreasing order of “importance” -in the sense of “number of cities”- of provinces (in BE, BG, and IT) or departments (in FR); the best function fit, Eq (7), is shown; parameter values are found in Table 2.

The function in Eq (7) is shown on Fig 7 to describe different cases, with various orders of magnitude, i.e., a semi-log plot of the number of cities in a province, *N*_*c*,*p*_ or in a department, *N*_*c*,*d*_, ranked by decreasing order of “importance”, for various countries (BE, BG, FR, IT). The reference year is 2011. In such cases, *ϕ* ≡ 0, obviously, thereby much simplifying Eq (8), whence reducing the fit to a three free parameter search.

For completeness, the main statistical indicators for the number of cities (*N*_*c*_), in the provinces (*N*_*c*,*p*_), regions (*N*_*c*,*r*_) or departments (*N*_*c*,*d*_) in these (European) countries, in 2011 is given in Table 2. Notice that the distributions differ: the median (*m*) is sometimes larger (or smaller) than the mean (*μ*), while the kurtosis and skewness can be positive or negative. Yet the fits with Eq (7) seem very fine. The large variety in these characteristics is an *a posteriori* argument in favor of having examined so many cases.

**Table 2:** Statistical characteristics of the distribution of the Number of cities *N*_*c*_, number of provinces *N*_*p*_ or departments, *N*_*d*_ (in FR), in 2011, in 4 European countries; relevant fit exponents with Eq (5), and entropic distance *d*.

	BE	BG	FR	IT
*N*_*c*_	589	264	36683	8092
*N*_*x*_ (*x* = *p*, *d*)	11	28	101	110
Min	19	10	1	6
Max	84	22	895	315
Mean (*μ*)	53.55	9.429	363.2	73.56
Median	64	10	332	60
Std Dev (*σ*)	20.32	4.273	198.3	55.34
Skewness	-0.311	0.781	0.332	1.729
Kurtosis	-1.045	1.480	-0.286	3.683
*μ*/*σ*	2.635	2.207	1.832	1.329
3(*μ* − *m*)/*σ*	-1.543	-0.401	0.472	0.735
*N*	11	28	101	110
*κ*_5_	7.49	10.28	84.69	203.8
*γ*	-0.160	0.157	0.133	0.386
*ξ*	0.631	0.310	0.653	0.529
Ψ	-0.0399	-0.985	-0.999	0.640
Φ	17.56	-0.820	0.265	0.906
*R*^2^	0.958	0.975	0.990	0.996
*ln*(*N*_*x*_) (*x* = *p*, *d*)	2.3979	3.3322	4.6151	4.7005
*d*	0.1587	0.1959	0.3793	0.2451

### 2.3 Modelization

The presented argument is of wide application as the reader can appreciate. However, the vocabulary in this modeling section can be adequately taken from the jargon of city evolution for better phrasing and for continuing with the analyzed data.

A preferential attachment process can be defined as a settlement procedure in urn theory, where additional balls are added and distributed continuously to the urns (areas, in this model) composing the system. The rule of such an addition follows an increasing function of the number of the balls already contained in the urns.

In general, such a process contemplates also the creation of new urns. In such a general framework, this model is associated to the Yule-Simon distribution, whose density function *f* is

f(a;b)=bB(a,b+1), (10)

being *a* and *b* real nonnegative numbers.

The integral $\int_{0}^{1}x^{a}\;{\left(1-x\right)}^{b}\;dx$ represents the probability of selecting *a* + *b* + 1 real numbers such that the first one coincides with *x*, from the second to the *a* + 1-th one numbers are less or equal to *x* and the remaining *b* numbers belong to [*x*, 1].

In practical words, newly created urn starts out with *k*_0_ balls and further balls are added to urns at a rate proportional to the number *k* that they already have plus a constant *a* ≥ −*k*_0_. With these definitions, the fraction *P*(*k*) of urns (areas) having *k* balls (cities) in the limit of long time is given by

$$P\left(k\right)=\frac{B(k+a;b)}{B(k_{0}+a;b-1)}$$ (11)

for *k* ≥ 0 (and zero otherwise). In such a limit, the preferential attachment process generates a “long-tailed” distribution following a hyperbolic (Pareto) distribution, i.e. power law, in its tail.

It is important to note that the hypothesis of continuously increasing urns is purely speculative, even if it is widely adopted in statistical physics. Indeed, such an assumption contrasts with the availability of resources, and the growth of the number of settlements is then bounded. Therefore, as in Verhulst’s modification [46] of the Keynesian expansion model of population, a “capacity factor” must be introduced in the original Yule process, thereby leading to the *u* term in Eq (5) and its subsequent interpretation.

## Entropy connection

One can consider to have access to a sort of “probability” for finding a certain “state” (size occurrence) at a certain rank, through

$$p\left(w\right)∼y_{2}\left(w\right)∼\frac{{\left(\phi +w\right)}^{-\zeta }{\left(1-w+\psi \right)}^{\chi }}{{\left(1+\phi +\psi \right)}^{\chi -\zeta +1}B\left(\chi +1,1-\zeta \right)}\;,$$

the denominator resulting from Eq (8).

Thereafter, one can obtain something which looks like the Shannon entropy [47]: S ≡ − *∫ p*(*w*) *ln*(*p*(*w*)). It has to be compared to the maximum disorder number, i.e. *ln*(*N*). Whence we define the relative distance to the maximum entropy as

$$d=\frac{S}{ln(N)}-1.$$ (12)

As a illustration, the only case of the ranking of cities in various countries is discussed. Values are reported in Table 2. It is observed that the FR and IT *d*-values are more extreme than those of BG and BE. This corroborates the common knowledge that the former two countries have too many cities, in contrast to the latter two.

Thus, in this particular case, this distance concept based on the universal ranking function with the two exponents *ζ* and *χ* shows its interest, e.g. within some management or control process. It can be conjectured without much debate that this concept can be applied in many other cases.

It is relevant to note that the entropy argument can be extended in a natural way to the *q*-Tsallis statistics analysis. Such an extension could add further elements to the thermodynamic interpretation of the proposed rank-size analysis. More in details, rank-size law might be associated to *q*-Tsallis distribution through a generalization of the central limit theorem for a class of non independent random variables (see e.g. [48] and [49]). However, the Tsallis approach is well-beyond the aim of the present study, and we leave this issue to future research.

## 3 Conclusions

This paper provides a basically three parameter function for the rank-size rule, based on preferential attachment considerations and strict input of finite size sampling. The analysis of the distribution of municipalities in regions or departments has proven the function value after its mapping into “dimensionless variables”. It seems obvious that the approach is very general and not limited to this sort of data. Other aspects suggest to work on theoretical improvements of the rank-size law connections, through ties with thermodynamics features, e.g., entropy and time-dependent evolution equations ideas.
